# Pressure-Volume Relationships in the Spinal Canal and Potential Neurological Complications After Epidural Fluid Injections

**DOI:** 10.3389/fpain.2022.884277

**Published:** 2022-07-07

**Authors:** Hemmo Bosscher

**Affiliations:** Texas Tech University Health Sciences Center (TTU HSC), Department of Anesthesiology and Cell Biology/Biochemistry, Lubbock, TX, United States

**Keywords:** spinal injection, epidural, subarachnoid, intracranial pressure, neurological complications, epiduroscopy, high volume

## Abstract

High-volume fluid injections into the spinal canal may lead to severe neurological complications. But when anatomical or pathological conditions in the spinal canal are unfavorable, even small volume epidural injections can cause dangerously high epidural, subarachnoid, and intracranial pressures or pressure gradients. Data obtained from the scientific literature and direct clinical observation are used to derive a first-order approximation of epidural, subarachnoid, and intracranial pressure responses to epidural fluid injections. Maximum allowable fluid volumes for single or multiple divided fluid injections over time are calculated. In the presence of spinal pathology, 10 ml of fluids may increase epidural pressure to >100 mmHg. Injection speed >4 ml per second may also generate dangerously high intraspinal and intracranial pressures. Intermitted bolus injections provide limited protection, but intraspinal pressures may rise very fast when a critical total injected volume is reached. Potential complications of increased intracranial pressures or large pressure waves include nerve palsies, tinnitus, blindness, stroke, and death. Spinal injections or endoscopy should be performed in an awake responsive patient or with direct cerebrospinal fluid pressure monitoring. A set of guidelines for epidural fluid management is given.

## Introduction

Low back pain is one of the most common complaints in the doctor's office. Treatment of low back pain has become a multibillion-dollar industry. While most methods of treatment are relatively safe, some have the potential for serious complications. Interventional pain management of low back pain often involves injection of fluid into the spinal canal. Injected fluids contain pharmacological agents such as local anesthetics or glucocorticosteroids and most practitioners are aware of the array of potential complications, but the effect of volume and speed of intraspinal injections on epidural pressure receives relatively little attention. However, incorrect epidural volume management, for example, during epiduroscopy, may lead to devastating consequences such as stroke or death. Considering the large number of spinal injections performed daily, basic knowledge of the mechanics of epidural fluid injections may increase awareness of the possibility of large intraspinal pressure increases after epidural fluid injections and may guide how to perform spinal injections safely.

The effects of spinal injections on epidural, subarachnoid, and intracranial pressure have been reported by several investigators. It is clear that pressure-volume relationship in the spinal canal varies markedly from person to person and depends strongly on spinal anatomy and pathology. In the following analysis, an attempt is made to derive a first-order approximation of the pressure response to epidural fluid injections. In the mathematical expressions, the compliance of the spinal canal and the extent of fluid leakage are represented by the patient-dependent constants A and B, respectively. Values of these constants can be estimated from scientific reports in the literature and clinical observation. Low values of A and B suggest the possibility of a large pressure increase even when small volumes are injected. While the first part of this discussion, e*pidural pressure and epidural fluid injections*, provides the scientific foundation for later sections, for readers less interested in mathematics, it may be passed over without losing clinical context. The second part, *application to epidural fluid injections*, summarizes the application of the mathematical analysis to medical practice. The third part, *effects of increased epidural and subarachnoid pressure*, gives a short review of neurological complications that may occur after high volume or rapid epidural fluid injections are reported.

## Epidural Pressure and Epidural Fluid Injections

Injection of a certain amount of fluid in the epidural space will increase epidural pressure (P_epi_). Change of pressure depends on the rate of injection, epidural volume, amount of fluid leaking from the epidural space, and compliance of the epidural space. Although these variables may be used in mathematical modeling, compartmentalization of the epidural space, strong dependence of the compliance on effective epidural volume, and variable egress of fluids make an accurate prediction of epidural pressures difficult. However, a qualitative analysis combined with clinical and experimental data may be sufficient for this discussion.

Under normal conditions, subarachnoid pressure is greater than epidural pressure, the difference being a function of the elasticity of the meningeal membranes separating the two compartments (1). Epidural pressure may not be the same throughout the epidural space. The presence of fibrous membranes, fat tissue, and bony indentations may compartmentalize the epidural space causing regional pressure differences. Intercompartmental pressure differences will result in fluid flow between compartments, depending on resistance to flow. Thus, injection of a volume (V_inj_) of fluids into a sub-compartment of the epidural space will increase pressure in that compartment. This pressure increase will in turn result in outflow (V_egress_) of fluids from that compartment into secondary compartments in or around the spinal canal. If one defines the resultant change in volume of the primary epidural compartment after a fluid injection as the effective volume change (ΔV_eff_), then


(1) $$\begin{array}{l} \Delta {\text{V}}_{\text{eff}}={\text{V}}_{\text{inj}}-{\text{V}}_{\text{egress}} \end{array}$$

A subsequent increase in the (effective) epidural pressure (P_epi_) following the injection is dependent upon the elasticity of the walls of the primary compartment. The relation between the change ΔV_eff_ and ΔP_epi_ can be expressed as the *compliance* (C_epi_,_eff_) of the effective epidural space:


(2) $$\begin{array}{l} {\text{C}}_{\text{epi},\text{eff}}=\Delta {\text{V}}_{\text{eff}}/\Delta {\text{P}}_{\text{epi}} \end{array}$$

which is not a constant, but a non-linear function of volume and pressure. Combining Equations (1), (2) yields:


(3) $$\begin{array}{l} \Delta {\text{P}}_{\text{epi}}=\left({\text{V}}_{\text{inj}}-{\text{V}}_{\text{egress}}\right)/{\text{C}}_{\text{epi},\text{eff}} \end{array}$$

where, at a given pressure, the change in compartmental pressure is directly proportional to the effective volume change (accumulated volume in the primary compartment) and inversely proportional to the compliance of that compartment.

This simple formula immediately provides some insight into epidural fluid dynamics. A large enough injected volume will lead to increased epidural pressure. However, V_egress_ may be large and buffer the injected volume keeping the effective epidural volume low and therefore keeping epidural pressure low even when larger fluid volumes are injected in the spinal canal. V_egress_ depends on the location of the needle or epiduroscope tip in the spinal canal. In the sacral epidural space, V_egress_ is large and large volumes can be injected relatively safely. V_egress_ of the thoracolumbar epidural space is much smaller, requiring more judicious fluid administration to avoid potentially dangerous pressure changes. V_egress_ also differs among animal species being studied. For example, V_egress_ in sheep is very low so that a small injected volume rapidly increases epidural pressures and subarachnoid pressures, resulting in syncope. In pigs, on the other hand, V_egress_ is large so that marked increases in epidural pressure do not readily occur (2). The human spine is an intermediate between these extremes. In humans, initial fluid injections are absorbed by secondary compartments. However, as the compartments into which fluid escapes become more and more saturated, the driving force for fluid dissipation falls and the V_egress_ may decrease markedly.

**Figure 1:**
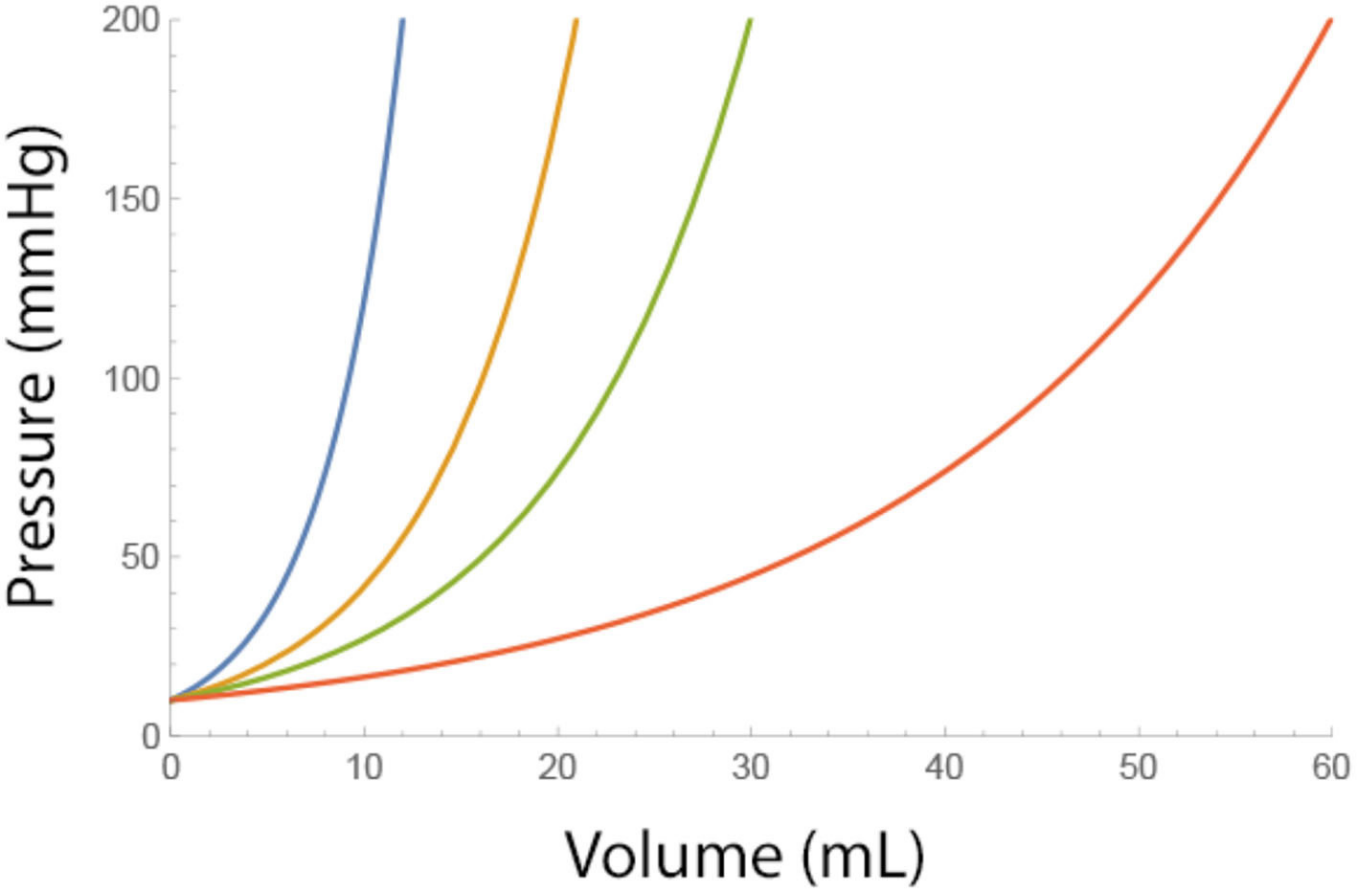
Effect of a single bolus fluid injection on the epidural pressure for different adverse conditions (i.e., low values of A). Blue A = 4, Brown A = 7, Green A = 10, and Red A = 20 ml. Pbaseline = 10 mmHg.

Effective epidural volume and effective compliance are difficult to determine, but the total injected fluid V_total_ is always known. The effect of increasing total injected volume on epidural pressure in the primary compartment can be deduced from the following approximation:


(4a) $$\begin{array}{l} \text{d}{\text{V}}_{\text{total}}/\text{d}{\text{P}}_{\text{epi}}\approx \text{A}/{\text{P}}_{\text{epi}} \end{array}$$

where dV_total_ is the change of total injected volume relative to the epidural pressure in the primary compartment. Here, A is considered constant in a neighborhood of P_epi_ and absorbs the effect of the anatomy and elasticity of the boundaries of the *entire* system of extra-dural compartments. Although a first-order approximation, the formula represents the important effect of increasing pressure on epidural compliance. The compliance as defined here is a (hyperbolic) function of pressure rather than volume because the pressure is measurable, constant over the geometrically complex anatomy of a sub-compartment, and ultimately the force per unit of area that leads to compression of epidural veins and soft tissues effecting a change in the buffering capability of the spine. In this approximation, compression of soft tissues and the shift of fluids between compartments decrease total epidural compliance asymptotically to zero. Therefore (by solving 4a), epidural pressure increases exponentially with additional fluid injections:


(4b) $$\begin{array}{l} {\text{P}}_{\text{epi}}\approx {\text{P}}_{\text{baseline}}*{\text{e}}^{\text{Vtotal}/\text{A}} \end{array}$$

where P_baseline_ is the epidural pressure before fluids are injected. As can be seen from this equation, low values of A and higher baseline pressures cause a steeper incline of epidural pressure (Figure 1).

An increase of pressure in one compartment leads to leakage of fluids into a secondary compartment having a lower pressure. If dP_epi_/dt is the rate of pressure change after a fluid injection in the primary compartment because of fluid leak, then the rate of epidural pressure drop is approximately a function of total volume injected and of epidural pressure itself, as given below:


(5a) $$\begin{array}{l} \text{d}{\text{P}}_{\text{epi}}/\text{dt}\approx -\text{B}*{\text{P}}_{\text{epi}}/{\text{V}}_{\text{total}} \end{array}$$

**Figure 2:**
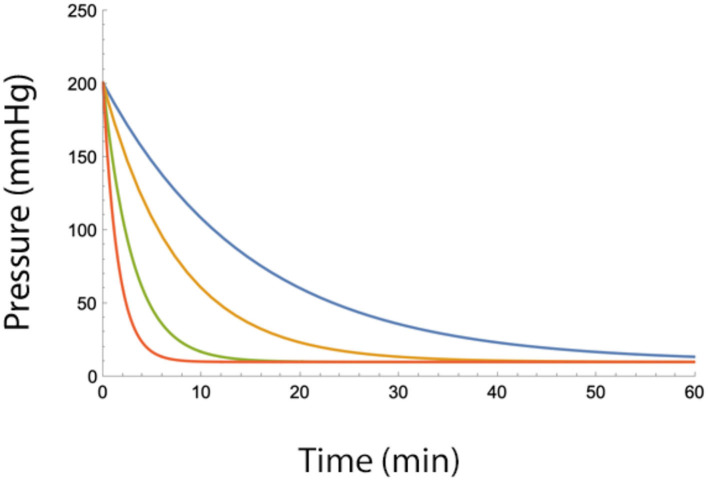
Epidural pressure as a function of time after injection of a bolus of fluid in the epidural space for different fluid egress conditions (i.e., low values of B). Blue B = 2, Yellow B = 4, Green B = 7, and Red B = 10 (ml/min). Total injected volume is 20 ml, A = 10 ml, Pbaseline = 10 mmHg.

The independent variable t is the time after the injection (min). B (ml/min) is considered constant in a neighborhood of P_epi_ and depends on the elasticity of the boundaries of the sub-compartments and on the resistance to flow between compartments, but is independent of the total injected volume. The relation shows that as secondary compartments fill up and egress of fluids from the primary compartment decrease, the rate of pressure drop in the primary compartment decreases asymptotically. Solving Equation 5a, the change of epidural pressure with time (t) is:


(5b) $$\begin{array}{l} {\text{P}}_{\text{epi}}\left(\text{t}\right)\approx {\text{P}}_{\text{epi},\text{max}}*{\text{e}}^{-\text{B }*\;\text{t}/\text{Vtotal}}\;+\;{\text{P}}_{\text{baseline}} \end{array}$$

where P_epi, max_ is the maximum epidural pressure change after a fluid injection. As can be seen from the equation, the epidural pressure decreases slower with low values of B (Figure 2).

Thus, without knowing the effective epidural volume and effective epidural compliance explicitly, Equations 4b, 5b can be combined to yield a relation between epidural pressure and total injected volume and time:


(6) $$\begin{array}{l} {\text{P}}_{\text{epi}}\approx {\text{P}}_{\text{baseline}}*\left(\text{1}+{\text{e}}^{\text{Vtotal}/\text{A}-\text{B}*\text{t}/\text{Vtotal}}\;-\;{\text{e}}^{-\text{B }*\;\text{t}/\text{Vtotal}}\right) \end{array}$$

**Figure 3:**
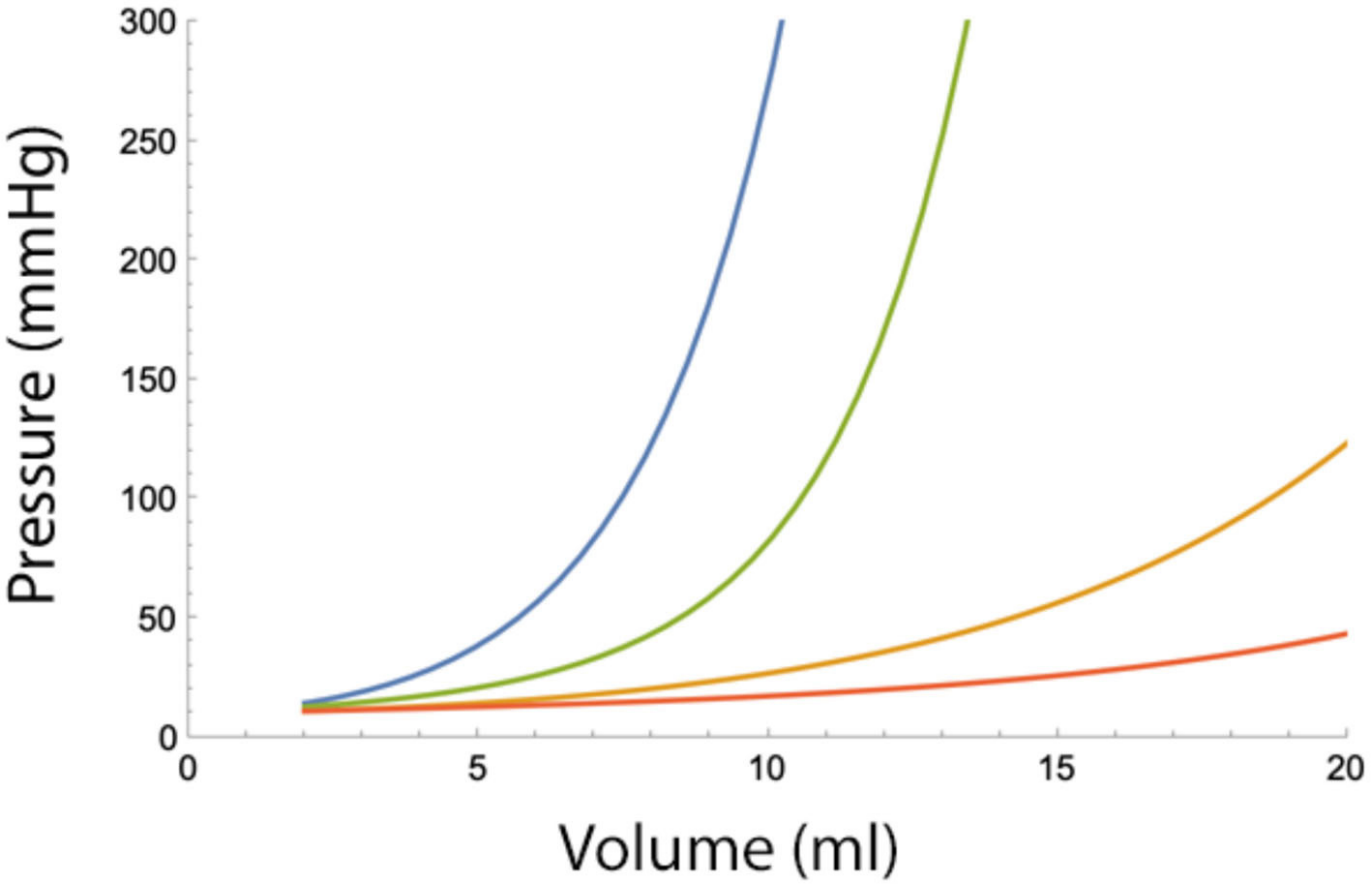
Epidural pressure after two epidural fluid bolus injections, 1 or 5 min apart, for severe (i.e., A = 4 ml and B = 4 ml/min) or moderately severe adverse conditions (i.e., A = 10 ml and B = 10 ml/min). Blue−1 min apart, severe conditions. Green−5 min apart, severe conditions. Yellow−1 min apart, moderately severe conditions. Red−5 min apart and moderately severe conditions.

Thus, A is a rough measure of the ability of the spinal canal to buffer epidural pressure against large fluid injections. Low values of A will lead to a more rapid increase in epidural pressure and a potentially elevated intracranial pressure (ICP). B is a measure of fluid leak from the primary compartment. A low value of B will diminish the mitigating effect of time on epidural pressure increases and causes a potentially sustained increase of ICP (Figure 3).

The effect of dividing a total injected volume into *n* multiple smaller injections can be calculated exactly. Assuming that fluid boluses V_total_/*n* and time intervals between injections T/*n* are constant, these calculations produce a rather convoluted equation:


(7a) $$\begin{array}{l} \;P\;\left(V,T,n\right)=P0*Exp\left[V/\left(A*n\right)\right]*\\ {}*(1+\underset{i=1}{\sum^{n}}(Exp\left[i*V/\left(A*n\right)-B*T/V*\left(\underset{j=n-i+1}{\sum^{n}}1/j\right)\right]\\ -Exp\left[\left(i-1\right)*V/\left(A*n\right)-B*T/V*\left(\underset{j=n-i+1}{\sum^{n}}1/j\right)\right])) \end{array}$$

Fortunately, for larger *n*, this can be approximated by


(7b) $$\begin{array}{l} {\text{P}}_{\text{epi}}\approx {\text{P}}_{\text{baseline}}*{\text{e}}^{\text{Vtotal}/(\text{A }*\text{ n})}(\text{1}-{\text{e}}^{\text{Vtotal}/(\text{A }*\text{ n})-\text{B }*\text{ T}/\text{ Vtotal}}\\ \;+\;{\text{e}}^{\text{2Vtotal}/\text{A}-\text{ 3B }*\text{ T}/\text{Vtotal}}) \end{array}$$

**Figure 4:**
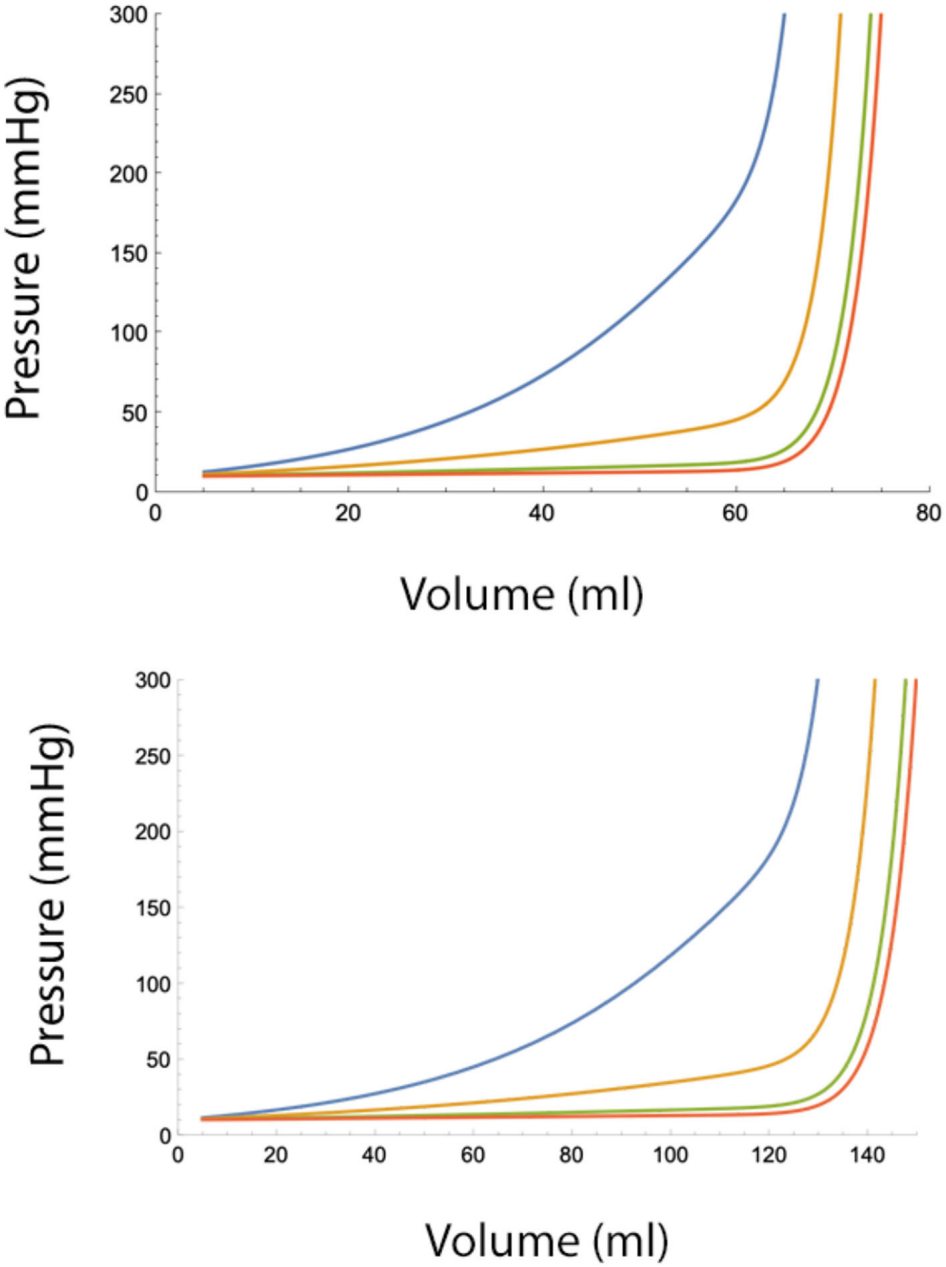
**(Top)** Epidural pressure after epidural fluid injection over 30 min under moderately severe adverse conditions (A = 10 ml, B = 10 ml/min). Injected volume is divided in *n* smaller aliquots, where *n* = 2 (blue), *n* = 4 (yellow), *n* = 10 (green), and *n* = 20. Pbaseline = 10 mmHg. **(Bottom)** Epidural pressure after epidural injection over 30 min under normal conditions (A = 20 ml, B = 20 ml/min). Injected volume is divided in *n* smaller aliquots, where *n* = 2 (blue), *n* = 4 (yellow), *n* = 10 (green), and *n* = 20 (red). Pbaseline = 10 mmHg.

Using mathematical software (e.g., Mathematica v12 [2021], Wolfram Research, Inc., Champaign, IL, USA), it can be shown that injecting the total volume in smaller aliquots is effective in controlling epidural pressures, but only to a limit. For any combinations of A and B, there exist a critical volume at which this protective effect disappears and epidural pressure rises rapidly (Figures 4A,B).

The term B^*^T/V_total_ in Equation 7b decreases with a rate 1/V_total_, while the term containing A increases with increasing V_total_ and *n*. From this, it can be seen that after a certain injected volume, the term containing A in Equation 7b dominates the terms containing B. At this critical value, additional fluid injections will cause a sudden and very fast rise in the pressure in the epidural space. Therefore, spreading injections over time provides only limited protection against large epidural pressure increases. It also can be seen that beyond a certain number, increasing the number of injections per time interval has a very little additional effect on epidural pressure reduction. This also implies that continuous infusion of fluid into the epidural space (i.e., *n* approaches ∞) offers no advantage over multiple small bolus injections.

Of course, over longer periods, fluids will be absorbed from secondary compartments, but this process may be very slow so that increases in epidural pressure can be sustained for extended periods of time.

Eventually, if the conditions in the epidural space are unfavorable, that is, limited volume buffering capacity of the epidural space (a low value of A) and/or high resistance to outflow from the epidural space (a low value of B) or if epidural fluid infusion (V_total_) becomes excessive, epidural pressure will rise. The trans-dural pressure gradient decreases until equilibrium between subarachnoid and epidural pressures has been achieved, at which point, the tensile elastic forces of the dura mater and arachnoid cease to create a pressure barrier between the epidural and subarachnoid space.

## Subarachnoid Pressure and Epidural Fluid Injections

Since at equilibrium, epidural pressure P_epi_ and subarachnoid pressure P_sas_ are the same (P_epi_ = P_sas_), the epidural and subarachnoid distribution of an injected volume (V_inj_) in the spinal canal can be calculated from the ratio of epidural to subarachnoid compliance, C_epi_/C_sas_ = ΔV_epi_/ΔV_sas_, and noticing that V_inj_ = ΔV_total_ = ΔV_epi_+ΔV_sas_:


(8) $$\begin{array}{l} \Delta {\text{V}}_{\text{sas}}={\text{V}}_{\text{inj}}/\left({\text{C}}_{\text{epi}}/{\text{C}}_{\text{sas}}+\text{1}\right) \end{array}$$

Where C_epi_ is the compliance of the entire system of extra-dural compartments. This can be used to calculate the change in subarachnoid pressure:


(9) $$\begin{array}{l} \Delta {\text{P}}_{\text{sas}}={\text{V}}_{\text{inj}}/\left({\text{C}}_{\text{epi}}+{\text{C}}_{\text{sas}}\right) \end{array}$$

V_sas_ is important because even though cerebrospinal fluid (CSF) volume may remain constant, increased epidural pressure causes fluid shifts to more compliant regions of the subarachnoid space such as the sacral and cervical subarachnoid space or the cerebral ventricular system, and may be as large as 30 ml under normal conditions. This fluid shift may initially mitigate subarachnoid pressure increases, but conditions will change rapidly when this buffer system is exhausted. At this point, compliance of the subarachnoid space will become much lower than the compliance of the epidural space so that:


(10) $$\begin{array}{l} \Delta {\text{P}}_{\text{sas}}\approx {\text{V}}_{\text{inj}}/{\text{C}}_{\text{epi}} \end{array}$$

Hence, subarachnoid pressure will become almost entirely dependent on injected volume and epidural compliance. If under certain adverse conditions, epidural compliance is also low, the addition of small fluid volumes into the epidural space will cause marked increases in subarachnoid pressure and therefore intracranial pressure. Finally, upon the substitution of P_sas_ for P_epi_ in Equations 4, 5:


(11) $$\begin{array}{l} {\text{P}}_{\text{sas}}\approx {\text{P}}_{\text{baseline }}*\left({\text{e}}^{\text{Vtotal}/\text{A}-\text{B }*\;\text{t}/\text{Vtotal}}\;-\;{\text{e}}^{-\text{B }*\;\text{t}/\text{Vtotal}}\;+\text{1}\right) \end{array}$$

It is of note that the constant A in this equation is the same (except for a factor log10) as the pressure-volume index (PVI) for subarachnoid fluid injections as derived by Marmarou et al. (3, 4). B represents the time constant of pressure decay after an injection (the inverse of the product of effective epidural compliance and outflow resistance) and is also consistent with the equation for outflow resistance as derived by Marmarou. Since the PVI for an individual patient is constant over a large pressure range, this suggests that A and B are also constant over a relatively large range of pressures.

With the increasing total volume and the diminishing effect of spreading fluid injections over time, the term B ${\;}_{{\;}^{*}}$ t/V_total_ vanishes and


(12) $$\begin{array}{l} {\text{P}}_{\text{icp}}={\text{P}}_{\text{sas}}\approx {\text{P}}_{\text{baseline}}*\left({\text{e}}^{\text{Vtotal}/\text{A}}+\text{1}\right) \end{array}$$

that is, intracranial pressure (P_icp_) increases *exponentially* with total injected volume.

## Pressure Waves and Epidural Fluid Injections

Large and sustained elevations of ICP may cause serious complications, but short-lasting high-amplitude pressure waves can also have a damaging effect on the delicate intraspinal and intracranial neural structures. The magnitude of the pressure generated at the tip of the epiduroscope (or needle) can be considerable after rapid fluid injections and depends on the geometry and compliance of the compartment in which fluid is injected. Bernoulli's equation, (1/2 ρ V^2^ + Δp= Constant), relates the pressure and fluid density (ρ) to the velocity (V) of the fluid at the tip of the needle or epiduroscope. This makes the speed of injection through the side port the determining factor of pressure generated at the tip of the epiduroscope, not force (although a larger syringe requires more force to generate the same speed). The effect of jet flow from the tip of the needle or epiduroscope into complex epidural geometry on epidural pressure is difficult to calculate but may be approximated if one assumes that the pressure wave is transmitted as a spherical wave from a point source. The amplitude of a spherical wave from a point source can be expressed as the sum of a series of sine waves:


(13) $$\begin{array}{l} \text{U}\left(\eta \left(\text{n}\right)\right)=\text{D}/\text{r}*\text{cos}\eta \left(\text{n}\right)\;\eta \left(\text{n}\right)=\kappa \left(\text{n}\right)*\text{r}-\text{c}*\text{t} \end{array}$$

Where U (η(*n*)) is the amplitude of the wave, *r* is the distance from the tip of the needle or epiduroscope, c is the speed of the wave, and t is time. κ (*n*) is the wave number of a component of the composite wave. η(*n*) is the independent variable for the nth sine wave component of the composite wave. D is a constant and depends on the physical properties of the fluid and the geometry of the anatomical structures involved.

Thus, the amplitude of a composite wave diminishes inversed proportionally with the distance from the point source. Dispersion of a composite wave will also decrease the amplitude. Therefore, in most instances, very high initial pressure waves at the epiduroscope or needle tip dissipate rapidly and may not generate significant forces on tissues. However, under certain conditions, the non-linearity of waves may balance dispersion and generate a *solitary wave or soliton* (e.g., a tidal wave). If the dural sac is considered to be a fluid-filled tube, such a pressure wave can be described mathematically as:


(14) $$\begin{array}{lll} P\left(\eta \right) & = & P_{\text{max}}*\text{sec}{\text{h}}^{\text{2}}\left(\;\eta /\text{W}\right),\\ \eta & = & \left(\text{X}-\text{c}*\text{t}\right),\\ P_{\text{max}} & = & \text{3c}/{\text{K}}_{\text{1}},\;\text{W}=\text{2}\surd \left({\text{K}}_{\text{2}}/\text{c}\right) \end{array}$$

where P_max_ is the maximum soliton amplitude at the dural sac generated by the injection, c is the speed of the wave, X is the distance over the length of the dura in the cranial direction, W is the width of the soliton, and t is time. K_1_ and K_2_ are constants depending on fluid properties and the geometry and physical properties of the anatomical structures involved. The independent variable η shows that the wave is stationary (5).

An important property of this type of wave for this discussion is that a solitary wave maintains its amplitude *without decay*. This has important implications for intraspinal fluid injections. Syringe pressure generated at the side port of the epiduroscope can be extremely high, while the pressure drop over the length of the needle or epiduroscope is negligible. It follows that a large pressure wave generated at the side port is transmitted virtually unchanged into the spinal canal. If the volume of the primary compartment in which the fluid is injected is small or if the epiduroscope tip is close to the dura mater, or worse, if the tip is accidentally placed in the subdural or subarachnoid space, large pressure waves may travel unaltered through the CSF toward the cranium where high pressures and steep pressure gradients of solitary waves may result in considerable damage to the delicate neural and vascular structures in the spinal canal and cranium. Notice that the width W of a soliton does not affect the amplitude of the wave, that is, small volumes injected at high speed may still generate damaging pressure waves in the central nervous system.

## Application to Fluid Management During Epiduroscopy

The above analysis of volume-pressure relationships is qualitative in the sense that the formulas suggest the global behavior of the system of spinal compartments, but do not accurately predict pressures over the whole range of injected volumes for all different geometries. In other words, the value of A, a measure of the pressure buffering capability of the epidural space, and the value of B, a measure of the speed at which fluids escape from the epidural space, are not constant over the entire range of pressures and depend on physical properties and the anatomy of the spinal canal. However, the objective of this discussion is to estimate maximal allowable fluid volumes that can be injected into the epidural space to ensure that the epidural pressure does not exceed a critical number. Therefore, only knowledge of the behavior of the system around a critical point is required. Over a small enough pressure interval, the values of A and B may be considered constant and can be calculated by inverting the above formulas and the use of known research and clinical data from the literature and clinical practice (Table 1). Of course, attempts to use these constants over the entire pressure range will result in errors, but for this discussion, the formulas may give reasonable estimates of the fluid volumes that can be safely injected into the epidural space.

**Table 1:** Values of A and B calculated from experimental data in the literature.

**Constants**	**A (ml)**	**B (ml/min)**
Usubiaga et al. (1)	4–10	7–14
Rocco et al. (6)	1–37	–
Hilt et al. (7)	10–14	4–17
Oh et al. (8)	9	–
Hirabayashi et al. (9)	–	10–30
Shapiro (10)	9–13*	–
Bosscher (clinical experience)	8**	2**

*Derived from the pressure volume index (PVI) calculated for the subarachnoid space. **Estimation derived from injected volumes at which some patients became symptomatic.

## Total Injected Volume

Neurological complications of spinal injections may occur when the subarachnoid pressure exceeds 20 mmHg and will become increasingly probable at a pressure >50 mmHg, especially when elevated pressures are sustained (11). From the table, it can be seen that the range of values of A derived from the pressure-volume index (PVI) in the subarachnoid space is similar to the values of A calculated from epidural volume-pressure relationships. This supports the interdependency of the epidural and subarachnoid compliance and the initial pressure buffering effect of subarachnoid fluid shifts. In most patients, a compliant epidural and subarachnoid space will keep intraspinal pressures within an acceptable range. Once compensatory mechanisms in the subarachnoid space are exhausted, baseline subarachnoid pressure will rise and CSF pressure depends now mostly on epidural compliance. Low epidural compliance may be found in areas of the central spinal canal and neural foraminal stenosis or areas of severe epidural fibrosis. Under these circumstances, subarachnoid pressures may rise rapidly with additional epidural fluid injections. The volume-pressure relationship of a *single* bolus injection depends on the value of A (Figure 1). The Maximum allowable *total* volume of multiple injections depends on both A and B. Critical volumes for a single bolus injection also depend on the baseline pressure. Table 2 gives critical volumes (ml) at which the epidural pressure will exceed 100 mmHg, for several baseline epidural pressures and low range values of A.

**Table 2:** Volumes (ml) of fluid bolus injections at which the epidural pressure exceeds 100 mmHg, for different baseline epidural pressures and values of A.

	**A (ml)**				
**Pbaseline (mmHg)**				
	**2**	**4**	**7**	**10**
5	6	11.5	22	30
10	4.5	9	16	23.5
15	3.5	8	13.5	19
20	3	6	11	16.5

From the table, it can be seen that values of A below 4 ml indicate high risk, 4–7 ml significant risk, 7–10 ml moderate risk, and values >10 a mildly increased risk for dangerously high epidural pressures. Under extreme conditions, the value of A can drop below 2, in which case, a 10-ml bolus injection may give epidural pressures well above 200 mmHg!

Normal values of B range from 10 to 20 ml/min but under adverse conditions can drop below 4 ml/min. The value of B becomes significant when the total injected volume is divided over several injections. Figure 3 shows the pressure-reducing effect of a waiting period between two bolus injections for several values of B. From the figure, it can be seen that under adverse conditions (low B), the pressure reduction is minimal. Dividing the total fluid volume over a larger number of smaller fluid injections reduces epidural pressure significantly. However, as can be seen in the figures, dividing the total fluid volume into 15 or more bolus injections over a 30-min interval does not provide much additional benefit (Figures 4A,B).

As can be seen from the graphs, epidural pressure remains well within acceptable range until a critical total volume has been injected. After this volume, additional fluid injections cause a sudden and very rapid increase in the epidural pressure (>200 mmHg). Table 3 gives the maximal allowable total fluid volume, divided into 15 epidural bolus injections, for epidural pressures not to exceed 100 mmHg for several values of A and B.

**Table 3:** Maximal allowable total fluid volume (ml), divided into 15 epidural bolus injections over 30 min, for epidural pressures not to exceed 100 mmHg for several values of A and B.

	**B (ml/min)**			
**A (ml)**				
	**2**	**4**	**10**	**20**
2	13	18	25	34
4	20	26	39	52
10	39	48	67	88
20	65	78	107	135

From the table, it can be seen that the general rule as suggested by Avellanal et al. (12) of a maximum total injected fluid volume of 60 ml over 60 min, may not keep the epidural pressure below 60 mmHg in all patients. Since adverse conditions cannot be reliably identified, the results of the above analysis suggest that some type of pressure or neuromonitoring such as electrophysiological response testing or direct communication with an awake and responsive patient is imperative. When a patient is heavily sedated or epiduroscopy is performed under general anesthesia and epidural or subarachnoid pressures are not known, only small amounts of fluid should be injected into the epidural space.

## Speed of Injection

The syringe pressure generated by pushing the plunger at different speeds for different syringes varies. No reports are available that relate syringe pressure to epidural and subarachnoid pressure. A rough estimation can be made by applying Bernoulli's Law. An injection speed of 5, 10, or 20 ml/s would raise the pressure at the tip of the epiduroscope to ~5, 20, and 80 mmHg, respectively. The maximum pressure generated by a syringe can be exceedingly high: >200 psi for a 3-ml syringe, >50 psi for a 10-ml syringe, and >20 psi for a 20-ml syringe (13). Thus, a 3- or 5-ml syringe injected in a fraction of a second may generate pressures >300 mmHg at the tip of the needle or epiduroscope. Although the amplitude of the pressure wave will decrease rapidly with the distance from the tip of the needle or epiduroscope, this reduction may be too small to prevent injury. Therefore, when conditions are unfavorable, fast injections can expose the spinal cord and brain to very high subarachnoid pressure waves that may cause significant damage. The constants that determine the shape of solitary waves are harder to derive but have some significance since destructive forces may not only depend on the absolute syringe pressure but also on the pressure differential, that is, small volumes injected at high speed may also cause damage.

The above analyses can be summarized as follows:

1. Subarachnoid pressure increase depends primarily on total injected volume. The epidural space provides only temporary protection against dangerously high intracranial pressures.
2. If epidural compliance is low (A is low), pressures may rise rapidly, even when small fluid volumes are injected into the epidural space.
3. If outflow resistance is high (B is low), pressure drops slowly, epidural pressure increases faster with subsequent injections, and elevated subarachnoid pressures are sustained.
4. Even when the capacity of a secondary compartment is large, epidural pressure may increase rapidly if outflow resistance is high and epidural compliance is low.
5. The effect of spreading fluid injections over time becomes negligible as the total injected volume increases.
6. Infusion of epidural fluids offers no advantage over bolus injections.
7. For intermitted injections, there exists a threshold volume after which epidural pressure increases abruptly.
8. If conditions are unfavorable (A is low, B is low), this threshold may be unexpectedly low and ICP may rise quickly to dangerously high levels, even when small amounts of fluid are injected into the epidural space.
9. Protective shifts of subarachnoid fluid shifts may be diminished when conditions that raise baseline intracranial pressure exists.
10. Fast fluid injections into the epidural space may cause steep pressure waves that are transmitted unaltered through the CSF and may exert significant forces on the tissues of the central nervous system even when baseline epidural and subarachnoid pressures are normal.
11. The pressure at the tip of the needle or epiduroscope is determined by the speed of injection, not syringe size.
12. Small volumes, injected at high speed into the epidural space, may cause significant damage to the central nervous system

## Effects of Increased Epidural Pressure

Markedly increased epidural pressure without an accompanying increase of ICP may occur when the contact surface between the epidural and subarachnoid space is small. For example, this situation may occur during a “lysis of epidural adhesions” procedure. High syringe pressures are transmitted virtually unaltered into the epidural space when the epiduroscope tip is placed in a small fibrotic compartment. Shear forces may rupture epidural blood vessels and nerve tissue. High normal forces may lead to sustained pressure on critical epidural blood vessels causing ischemia of the nerve roots of the cauda equina or the distal spinal cord resulting in neurological deficits (14). Indeed, transient urinary retention, radiculopathy, and transient paralysis have been reported with compartmental loculation of fluids in the epidural space.

In patients with epidural fibrosis after back surgery, injection of fluids in a small low compliant fibrotic compartment may also lead to rupture of the dura mater and arachnoid and inadvertent subdural or subarachnoid placement of the needle or epiduroscope. Subdural or subarachnoid placement may not always be obvious. The narrow subdural space is easily expanded by subsequent fluid injections and causes potentially damaging fluid shifts or solitary waves in the CSF. Rupture of the thin arachnoid will lead to an absolute increase in CSF volume and a rapid increase of ICP when such a placement is not immediately recognized and additional fluid injections continue. Inadvertent subdural or subarachnoid injections of local anesthetics may lead to cardiovascular instability, loss of consciousness, and respiratory arrest. Injection of hyperosmotic agents or neurolytics may lead to direct axonal damage.

## Effect of Increased Subarachnoid Pressure

The most important consequence of increased epidural pressure is increased subarachnoid and therefore increased intracranial pressure. Under favorable conditions, a relatively large amount of fluid can be injected into the epidural space without a significant rise in ICP. Unfortunately, routine diagnostic studies may not reliably identify unfavorable conditions. When epidural anatomy is altered, such as in patients with multilevel spinal stenosis or severe fibrosis after back surgery, ICP may rise rapidly even after injection of a small amount of fluid and cause serious complications (15). In patients considered to be low risk, excessive fluid injections may be used when visualization is poor or when extensive epidural procedures require prolonged and optimal exposure to epidural structures. Typical complaints of increased ICP are headache, neck and shoulder pain, nausea, and vision and hearing disturbance, such as blurred vision, tinnitus, and confusion.

Intracranial pressure may rise more rapidly when baseline ICP is increased. CSF turnover is well-regulated but may be altered in the presence of brain tumors, infections, cerebrovascular insufficiency or accident, or venous outflow obstruction from the cranium. Idiopathic increased intracranial hypertension should be suspected in obese women complaining of frequent headaches, nausea and blurred vision, and cognitive dysfunction (16). Ultimately, sustained increased ICP leads to damage to the ependyma and interstitial edema of the parenchyma of the brain and spinal cord. Decreased cerebral perfusion pressure, particularly in watershed areas, may lead to ischemia and neurological deficit. Venous compression and compensatory high arterial pressure may cause congestion and extravasation of blood causing microhemorrhages in the brain, spinal cord, nerve roots, and sensory organs. Rapid injection of epidural injections may cause dizziness, nausea, frontal oppression, contracture of the back muscles, and tachypnea. Strong pressure waves cause large pressure gradients over neural and vascular tissues and small but the forceful displacement of the brain tissues within the cranium exerts traction or pressure on blood vessels and cranial nerves. These forces may rupture thin-walled veins or small arteries and cause direct damage to neural tissue. Although the immediate effects of increased intracranial pressure are mostly transient, persistent parenchymal changes may lead to prolonged post-operative neurological dysfunction such as altered level of consciousness, seizures, and cognitive impairment (15).

Antegrade loss of memory after epiduroscopy has been reported (17). More subtle alterations of executive function are likely to occur after epiduroscopy but are often not addressed during routine post-operative neurological evaluation (18). Very high, sustained intracranial pressures or steep pressure gradients may cause cerebral edema, decreased cerebral perfusion pressure, or hemorrhagic stroke resulting in major neurological deficit or death.

### Loss of Vision

The optic nerve is partially contained in an extension of the arachnoid membrane. Raised ICP causes edema of optic nerve fibers blocking the fast and slow axoplasmic transmission in the optic nerve axons. In addition, increased ICP will compress the central retinal vein causing venous stasis and bleeding in the posterior segment of the eye. Extravasation is enhanced by a reflex increase of the ophthalmic artery pressure. Vision loss is usually transient but may be persistent if bleeding occurs within the optic nerve (19, 20). Large pressure waves may cause direct injury to pial bridging veins and render the posterior optic nerve ischemic. Rupture of larger veins or arteries may lead to more extensive bleeding in any of the ophthalmic structures and may cause permanent loss of vision (21, 22).

### Loss of Hearing and Equilibrium Disturbances

The subarachnoid space and inner ear intra-labyrinthine fluid communicate through three routes: the vestibular aqueduct, the cochlear aqueduct, and the internal auditory canal so that the vestibulocochlear pressures mirror subarachnoid pressures (23). Increased subarachnoid pressure and pressure differentials in perilymph and endolymph may cause direct damage to the delicate vascular and neural structures within the cochlea and labyrinth similar to the mechanisms described above (24). In addition, shearing and normal forces may cause dysfunction of the vestibuloacoustic nerve. Tinnitus, hearing loss, or equilibrium disturbances may be transient, but large pressure waves are likely to cause permanent loss of cochlear and vestibular function (19).

### Cranial Nerve Palsies

Cranial nerve palsies are common and may occur in 1/300–1/8,000 patients receiving spinal anesthesia. Cranial nerves are anatomical equivalent to the nerve roots in the spinal canal. The cranial nerves course within the intracranial subarachnoid space, as they pass toward the target tissues, and exposes them to the forces described earlier. The shift of the brain and brainstem in the cranium, large pressure waves in the CSF, or sustained increased intracranial pressure may cause stretching or compression of any of the cranial nerves. Direct axonal and vascular damage may cause temporary or permanent dysfunction of cranial nerves. Third, fourth, and sixth nerve palsies causing ophthalmoplegia and anisocoria have been reported after spinal or epidural injections (25–27). Trigeminal or facial nerve palsy may cause loss of facial sensation or facial muscle weakness (28, 29). Dysphagia may result from glossopharyngeal nerve dysfunction. Pharyngeal weakness and loss of taste after spinal injection are rare but may occur (30). Unilateral tongue atrophy as a result of the hyoglossus nerve injury has been reported (31). Most cranial nerve palsies are caused by low intracranial pressure associated with a dural puncture but may occur with large intracranial pressure changes. Fortunately, symptoms are mostly transient but may be permanent when cranial nerves are exposed to prolonged or excessive shear or normal forces.

## Preventative Actions

To minimize the risk of neurological deficit, the author has adopted a set of guidelines that may minimize the incidence of pressure-related complications of epidural fluid injections:

1. Inject the smallest amount of fluid needed at a time. Any fluid injection into the epidural space is potentially dangerous, even when small volumes are injected.
2. Without pressure monitoring, absolute volumes should be minimized and not exceed 60 ml unless epidural or subarachnoid pressure is monitored or sufficient fluid egress from the epidural space is established.
3. A single bolus injection should not exceed 10 ml.
4. As total injected volume increases, the number and volume of bolus injections should be reduced.
5. Be aware that after a certain critical total injected volume, additional injections may increase the epidural pressure abruptly to very high levels.
6. Small intermittent injections are recommended, but be aware that as the total amount of fluid injected in the epidural space increases, the degree of protection may become negligible.
7. Control speed of injection to <3–4 ml/s, independent of syringe size.
8. If risk factors such as epidural adhesions, multilevel spinal stenosis, or idiopathic increased intracranial pressure are present, epiduroscopy should be performed only if some degree of neurological or pressure monitoring is established.
9. Unfortunately, risk factors are often not identified and a seemingly compliant appearing epidural space on epiduroscopy is not a reliable finding.
10. A conscious responsive patient is desirable when spinal injections are performed. Epiduroscopy under general anesthesia should be avoided and if performed, with a reduced total injected volume or with pressure or neurophysiological monitoring.
11. If the patient becomes symptomatic after injection (e.g., complains of neck pain or headaches), the procedure should be aborted since a waiting period is unlikely to improve adverse conditions in the spinal canal.
12. For epiduroscopy, the 3 × 60 rule, limit total injected volume to 60 ml, limit the total duration of the procedure to <60 min, and avoid subarachnoid pressures of >60 mmHg, applies to most patients but is arbitrary and should not be generalized.
13. Absolute guidelines on epidural fluid management cannot be given, but in about 2,000 *mostly conscious* patients undergoing epiduroscopy, the author has routinely used 60–70 ml of normal saline and 20–30 ml of contrast material, local anesthetic, and treating medications, without neurological sequelae. However, some patients reported headaches and neck pain after injection of a small volume (<30 ml) of normal saline. These complaints diminished only after a considerable waiting time (>15 min).
14. Incorrect fluid management is currently an unrecognized but important cause of serious complications of intraspinal fluid injections. Procedures can be performed safely if one is aware of the dangers of epidural fluid injections, injections are performed with caution, and if some degree of direct or indirect monitoring of intraspinal pressures is maintained throughout the procedure (20).

## Data Availability Statement

The original contributions presented in the study are included in the article/supplementary material, further inquiries can be directed to the corresponding author/s.

## Ethics Statement

Ethical review and approval was not required for this study in accordance with the local legislation and institutional requirements.

## Author Contributions

The author confirms being the sole contributor of this work and has approved it for publication.

## Conflict of Interest

The author declares that the research was conducted in the absence of any commercial or financial relationships that could be construed as a potential conflict of interest.

## Publisher's Note

All claims expressed in this article are solely those of the authors and do not necessarily represent those of their affiliated organizations, or those of the publisher, the editors and the reviewers. Any product that may be evaluated in this article, or claim that may be made by its manufacturer, is not guaranteed or endorsed by the publisher.
